# Factors Influencing the Prescription of Cardiovascular Preventive Therapies in Patients with Peripheral Arterial Disease

**DOI:** 10.1371/journal.pone.0148069

**Published:** 2016-02-05

**Authors:** Myriam L. Montminy, Valerie Gauvin, Stephane Turcotte, Alain Milot, Yvan Douville, Isabelle Bairati

**Affiliations:** 1 Department of Vascular Surgery, Centre Hospitalier Universitaire de Québec, Quebec, Canada; 2 Research Center of the Centre Hospitalier Universitaire de Québec, Quebec, Canada; 3 Department of Vascular Medicine, Centre Hospitalier Universitaire de Québec, Canada; 4 Public Health Department, Centre Hospitalier Universitaire de Québec, Québec, Canada; Katholieke Universiteit Leuven, BELGIUM

## Abstract

**Background:**

Guidelines recommend that patients with peripheral arterial disease should be medically treated to reduce the occurrence of serious cardiovascular events. Despite these recommendations, studies conducted in the early 2000s reported that medical therapies for secondary cardiovascular prevention are not given systematically to patients with peripheral arterial disease (PAD). We identified factors associated with the prescription of preventive therapies in patients with symptomatic PAD.

**Methods and Findings:**

Consecutive patients with symptomatic peripheral arterial disease (n = 362) treated between 2008 and 2010 in one tertiary care center (CHU de Quebec, Canada) were considered. Data were collected from the medical charts. The main outcome was the combined prescription of three therapies: 1) statins, 2) antiplatelets, 3) angiotensin-converting-enzyme inhibitors or angiotensin receptor blockers. The mean age was 70 years and 43% had a pre-existing coronary artery disease. Antiplatelet therapy was the most prescribed drug (83%). A total of 52% of the patients received the three combined therapies. Less than 10% of patients had a known contraindication to one class of medication. Having at least three cardiovascular risk factors (Odds Ratio (OR) = 4.51; 95% CI: 2.76–7.37) was the factor most strongly associated with the prescription of the combined therapies. Pre-existing coronary artery disease (OR = 2.28; 95% CI: 1.43–3.65) and history of peripheral vascular surgery (OR = 2.30; 95% CI: 1.37–3.86) were two factors independently associated with the prescription of the combined therapies. However, peripheral arterial disease patients with chronic critical limb ischemia were less likely to receive the combined therapies (OR = 0.53; 95% CI: 0.32–0.87) than those with claudication. The retrospective nature of this study, not allowing for an exhaustive report of the contraindication to medication prescription, is the main limitation.

**Conclusion:**

About half of the patients with peripheral arterial disease were not optimally managed. Patients with multiple cardiovascular risk factors were more likely to receive the combined therapies. We still need to better understand the barriers and facilitators to the application of the guidelines.

## Introduction

Peripheral arterial disease (PAD) is a common condition, that affects up to 20% of individuals aged over 75 [1]. Patients with PAD have a marked increased risk of developing serious coronary and cerebrovascular events [2].

Clinical trials showed that medical management of PAD patients decreases the occurrence of major cardiovascular events both in patients with or without concomitant coronary artery disease [3–7]. A statin therapy was shown to be associated with a 22% relative reduction in the rate of the occurrence of first major vascular event, irrespectively of the LDL cholesterol levels [3]. A similar significant effect was also observed for patients with PAD but without pre-existing coronary disease. A meta-analysis conducted among 9214 patients with PAD showed a reduction of 23% in serious vascular events in those receiving an antiplatelet agent [5]. An angiotensin-converting-enzyme inhibitor (ACEI), ramipril, reduced by 22% the risk of myocardial infarction, stroke or death from cardiovascular causes in patients with high risk for cardiovascular events [6]. This beneficial effect was also observed in the sub-group of patients with PAD [4]. In addition, ramipril was shown to reduce cardiovascular events in both clinical and subclinical PAD. The beneficial effect of ACEI may be effective irrespective of its blood-pressure lowering effect. The ONTARGET study showed that the use of angiotensin receptor blocker (ARB) and that of ACEI were equivalent in reducing the occurrence of major cardiovascular event in PAD patients [8]. Moreover, the benefits of all these drugs appear to be independent, suggesting a cumulative risk reduction of about 75% [2].

As a result, guidelines, including a Canadian consensus published in 2005, state that there are benefits for all patients with PAD to receive a regimen including statins, antiplatelets, and ACEI in secondary prevention of major cardiovascular events [2,9–11]. In addition, the European guideline published in 2011 recognized the equivalence of ARB to ACEI [11]. However, studies conducted in the early 2000s reported that medical therapies for secondary cardiovascular prevention are not given systematically to patients with PAD [12–16].

To describe the management of PAD patients since the publication of these guidelines, we assessed the percentage of patients with PAD having a prescription for the recommended medical drugs in our institution at the time of their first femoropopliteal percutaneous transluminal angioplasty (PTA). We also identified factors associated with the prescription of the preventive combined therapies.

## Materials and Methods

### Study population

This study was conducted as part of a retrospective cohort study evaluating the prognosis of patients with PAD treated by a first femoropopliteal PTA. All referred patients with a suspected diagnosis of PAD had an ankle/brachial blood pressure test. The ankle/brachial index must be ≤ 0.90 for a PAD diagnosis. In addition, before any vascular intervention, PAD is documented by angiography. Eligible patients had to be treated consecutively between November, 1^st^ 2008 and December 31, 2010 at the CHU de Québec, St-François d’Assise Hospital, Quebec City, Canada. Patients with acute ischemia or coagulation disorders were ineligible. This study was approved by the Research Ethics Committee of the CHU de Québec. No consent was given because the data were analyzed anonymously.

### Data collection

All data were collected from patient’s medical charts using a standardized form. This form was designed to collect patient’s characteristics, medical and surgical histories, cardiovascular risk factors at the time of the referral, PAD characteristics (clinical symptomatology according to the Rutherford classification). In addition, we collected data concerning their most recent appointments for their PAD with family doctors, vascular surgeons or vascular internists, the context of the referral in vascular surgery (outpatient clinic vs hospital, emergency room vs not), and the characteristics of the medical doctor who did the reference (speciality, age, sex, years of practice). We also estimated the percentage of patients having the most frequently reported absolute and relative contraindications to each of the preventive therapies.

Social and material deprivation indexes were generated using the patient’s postal code to assess their socio-economic status [17]. The Charlson comorbidity index was calculated by scoring the comorbidities of the patients [18]. Pre-existing coronary artery disease (CAD) was defined by a previous history of myocardial infarction or coronary revascularisation. History of peripheral vascular surgery included all vascular surgeries, except those of the coronary arteries. An index of cardiovascular risk factors was constructed by adding the presence of four modifiable cardiovascular risk factors (smoking, diabetes, hypertension and dyslipidemia) varying between 0 and 4. Renal impairment was described according to KDOQI chronic kidney disease classification [19]. The characteristics of the referring physician were obtained using the online register of the medical practice board [20].

All patients had to provide, at the time of their vascular intervention, their latest drug list, which was integrated in their medical chart. We extracted all the medications of interest used at the time of the intervention, as well as their potential contraindications. As outcomes, we only considered preventive therapies known to reduce cardiovascular events in PAD patients with the highest level of evidence (level 1A) [2]. Based on the Canadian, American and European recommendations regarding the medical management of patients with PAD, our main outcome was the combined use of the following three therapies: 1) statins, 2) antiplatelets, 3) ACEI or ARB medications [2,9–11]. We also considered the use of each of the three therapies.

### Statistical analysis

Standard descriptive analyses were done to generate the percentages of patients receiving the recommended medications. Student t-tests and Chi-square tests were performed to identify potential factors associated with the prescription of each recommended therapy, as well as with the combined therapies. All the variables significantly associated with the prescription of the recommended therapies in the bivariate analyses (P< .05) were considered for inclusion in the multivariate logistic regression model [21]. In addition, we verified collinearities among the independent variables and excluded highly correlated variables. For each model, a stepwise procedure was used for selection of variables in the logistic regression. Adjusted odds ratios (OR) and their 95% confidence intervals (CI) were estimated. Statistical analyses were done using SAS, version 9.3 (SAS Institute, Cary, NC). All statistical tests were two-sided (α = .05).

## Results

The mean age of the 362 patients with PAD was 70 years (SD 10.8), 64% were men and the mean Charlson comorbidity index was 7.3 (SD 2.4) (Table 1). The mean of systolic blood pressure was 140.9 mmHg (SD = 17) and the mean of body mass index was 27.5 (SD = 5.4). Pre-existing CAD was present among 42.5% of the patients and diabetes among 39.8%. Critical limb ischemia (CLI) was the PAD symptomatology for 45% of the patients. About half (55.2%) of the patients had previous appointments with a vascular surgeon, while a minority (16.6%) had previous appointments with a vascular internist. Half of the referring physicians were family doctors.

**Table 1:** Patient’s demographics and clinical characteristics (n = 362).

Patient characteristics	
Age, mean (SD)	70 (10.8)
Male, n (%)	234 (64)
BMI, mean, (SD), kg/m2	27.5 (5.4)
Systolic arterial pressure, mean, (SD), mmHg	140.9 (17)
Diastolic arterial pressure, mean, (SD), mmHg	69.9 (8.3)
Charlson Comorbidity Index, mean (SD)	7.3 (2.4)
Smoking, n (%)	
None	54 (14.9)
Former	168 (46.4)
Current	140 (38.7)
Diabetes, n (%)	144 (39.8)
Hypertension, n (%)	291 (80.4)
Dyslipidemia, n (%)	270 (74.6)
Modifiable cardiovascular risk factors^a^, n (%)	
0–2 factors	198 (54.7)
3–4 factors	164 (45.3)
Renal impairment, n (%)	
Mild	134 (37)
Moderate	90 (24.9)
Severe	20 (5.5)
End-stage	3 (0.8)
History of vascular surgery, n (%)	107 (29.6)
Pre-existing coronary artery disease, n (%)	154 (42.5)
Ankle-brachial index, mean (SD)	0.60 (0.20)
Rutherford classification, n (%)	
Claudication	199 (55.0)
Critical limb ischemia	163 (45.0)
Family physician as the referring physician, n (%)	181 (50)
Previous appointments in vascular surgery, n (%)	200 (55.2)
Previous appointments in vascular medicine, n (%)	60 (16.6)
Previous appointments in family medicine, n (%)	351 (96.9)
Antiplatelets, n (%)	300 (82.9)
Statins, n (%)	264 (72.9)
ACEI, n (%)	143 (39.5)
ARB, n (%)	109 (30.1)
ACEI or ARB, n (%)	246 (68)

BMI, body mass index; ACEI, angiotensin-converting-enzyme inhibitor; ARB, angiotensin receptor blocker

^a^Included: current smoking, presence of diabetes, hypertension and/or dyslipidemia

**Fig 1:**
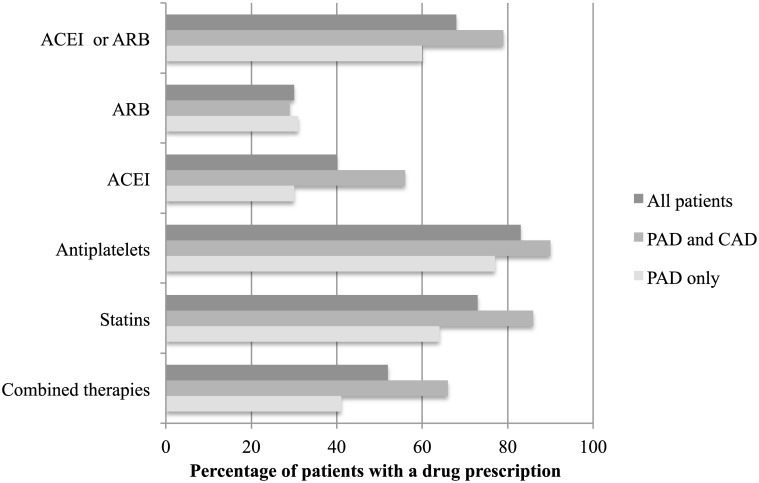
Prescription of the recommended therapy. ACEI, angiotensin-converting-enzyme inhibitor; ARB, angiotensin receptor blocker; CAD, coronary artery disease; PAD, peripheral arterial disease.

At the time of their vascular intervention, a total of 300 (83%) patients with PAD had a prescription of antiplatelets. Most of them (n = 264) received aspirin. Of these, 228 patients received aspirin at a low-dose (80 mg daily). Statins were prescribed in 73% of the patients, while ACEI or ARB was given to 68% of the patients (Fig 1). About half (52%) of the patients with PAD received the combined therapies. Patients with pre-existing CAD received more frequently the combined therapies than those without CAD (66.2% vs 41.3%, P< 0.0001).

A total of 38 patients (10.5%) had a known relative contraindication to antiplatelet therapies in their medical records (alcohol abuse, ulcer). Among these patients, 78% received an antiplatelet. Only one patient (0.2%) did not receive a statin because he had an acute liver disease, an absolute contraindication to statins, and this patient did not received statins. Among the 27 patients (7.5%) having a chronic liver conditions and/or a problem of alcohol abuse, 81% received a statin treatment. Ten patients (2.8%) had fibrate therapy, but half of them also received statins. With regard to ACEI or ARB, 23 patients (6.4%) had a severe or terminal renal insufficiency, but 74% of these patients received an ACEI or ARB.

Bivariate analyses showed that the factors consistently associated with the prescription of each of the therapeutic agent, as well as the combined therapies, were having several cardiovascular risk factors and having pre-existing CAD (Table 2). Patients receiving the therapies were significantly younger than those not receiving these treatments, except for ACEI/ARB. Having had previous appointments in vascular medicine or vascular surgery, as well having a history of peripheral vascular surgery were positively associated with the prescription of all the medications, except for ACEI/ARB. No significant association was found with the patient’s socioeconomic status and comorbidities.

**Table 2:** Factors associated with the prescription of the recommended therapies in bivariate analysis.

	Combined therapies			Statins			Antiplatelets			ACEI / ARB		
	Yes	No	P-value	Yes	No	P-value	Yes	No	P-value	Yes	No	P-value
Factors	n = 188	n = 174		n = 264	n = 98		n = 300	n = 62		n = 246	n = 116	
Age, mean (SD)	68.3 (9.2)	71.5 (12.0)	0.005	68.5 (9.6)	73.3 (12.8)	0.001	68.9 (10.5)	74.2 (11.3)	0.0005	69.8 (10.3)	69.9 (11.8)	0.90
Male, n (%)	129 (68.6)	105 (60.3)	0.10	181 (68.6)	53 (54.1)	0.01	198 (66)	36 (58.1)	0.23	159 (64.6)	75 (64.7)	0.99
Current smokers, n (%)	61 (32.4)	79 (45.4)	0.01	90 (34.1)	50 (51)	0.003	111 (37)	29 (46.8)	0.15	81 (32.9)	59 (50.9)	0.001
Diabetes, n (%)	102 (54.3)	42 (24.1)	<0.0001	121 (45.8)	23 (23.5)	0.0001	130 (43.3)	14 (22.6)	0.002	121 (49.2)	23 (19.8)	<0.0001
Hypertension, n (%)	172 (91.5)	119 (68.4)	<0.0001	222 (84.1)	69 (70.4)	0.004	243 (81)	48 (77.4)	0.52	226 (91.9)	65 (56.0)	<0.0001
Dyslipidemia, n (%)	170 (90.4)	100 (57.5)	<0.0001	235 (89.0)	35 (35.7)	<0.0001	24.2 (80.7)	28 (45.2)	<0.0001	196 (79.7)	74 (63.8)	0.001
Modifiable CV risk factors, n (%)^a^												
0–2	71 (19.6)	127 (35.1)	<0.0001	124 (34.3)	74 (20.4)	<0.0001	48 (13.3)	21 (5.8)	0.0002	28 (7.7)	41 (11.3)	<0.0001
3–4	117 (32.3)	47 (13)	<0.0001	140 (38.7)	24 (6.6)	<0.0001	103 (28.5)	26 (7.2)	0.0002	84 (23.2)	45 (12.4)	<0.0001
Renal impairment, n (%)	119 (63.3)	128 (73.6)	0.04	171 (64.8)	76 (77.6)	0.02	201 (67.0)	46 (74.2)	0.27	163 (66.3)	84 (72.4)	0.24
Pre-existing CAD, n (%)	10 (54.3)	52 (29.9)	<0.0001	132 (50.0)	22 (22.4)	<0.0001	139 (46.3)	15 (24.2)	0.001	122 (49.6)	32 (27.6)	<0.001
History of vascular surgery, n (%)	73 (38.8)	34 (19.5)	<0.0001	97 (36.7)	10 (10.2)	<0.0001	104 (34.7)	3 (4.8)	<0.0001	80 (32.5)	27 (23.3)	0.07
Critical limb ischemia, n (%)	82 (43.6)	81 (46.6)	0.58	106 (40.2)	57 (58.2)	0.002	131 (43.7)	32 (51.6)	0.25	112 (45.5)	51 (44.0)	0.78
Other antihypertensive drugs, n (%)	156 (83.0)	121 (69.5)	0.003	213 (80.7)	64 (65.3)	0.002	233 (77.7)	44 (71)	0.26	204 (82.9)	73 (62.9)	<0.0001
Reference via emergency room, n (%)	15 (8.0)	22 (12.6)	0.14	18 (6.8)	19 (19.4)	0.0005	32 (10.7)	5 (8.1)	0.54	21 (8.5)	16 (13.8)	0.12
Family physician as the referring physician, n (%)	78 (41.5)	103 (59.2)	0.0008	117 (44.3)	64 (65.3)	0.0004	137 (45.7)	44 (71.0)	0.0003	120 (48.8)	61 (52.6)	0.50
Previous appointments in												0.33
Vascular medicine, n (%)	41 (21.8)	19 (10.9)	0.005	56 (21.2)	4 (4.1)	<0.0001	60 (20)	0 (0)	<0.0001	44 (17.9)	16 (13.8)	0.33
Vascular surgery, n (%)	115 (61.2)	85 (48.9)	0.019	166 (62.9)	34 (34.7)	<0.0001	181 (60.3)	19 (30.6)	<0.0001	136 (55.3)	64 (55.2)	0.98

CV, cardiovascular; CAD, coronary artery disease.

^a^Included: current smoking, diabetes, hypertension, dyslipidemia

In the multivariate analysis, the factors positively associated with the use of the recommended combined therapies were having at least three cardiovascular risk factors (OR = 4.51; P< 0.0001), having pre-existing CAD (OR = 2.28; P = 0.0006), and having a history of peripheral vascular surgery (OR = 2.30; P = 0.002) (Table 3). Patients having critical limb ischemia rather than claudication had significantly lower odds of receiving the combined therapies (OR = 0.53; P = 0.01). Having pre-existing CAD and having at least three cardiovascular risk factors also remained significantly associated with the use of statins, antiplatelets or ACEI/ARB. Patients referred for the PTA by their family physician were less likely to received antiplatelets than those referred by a specialist (OR = 0.51; P = 0.04). In multivariate analyses, age was not an independent factor associated with the prescription of any of the therapies.

**Table 3:** Factors associated with the prescription of the recommended therapies in multivariate analysis.

Factors	Combined therapies	Statins	Antiplatelets	ACEI / ARB
	OR (95% CI); P-value	OR (95% CI); P-value	OR (95% CI); P-value	OR (95% CI); P-value
Rutherford classification				
Claudication	1	1	1	-
Critical Limb ischemia	0.53 (0.32–0.87); 0.01	0.25 (0.14–0.44); <0.0001	0.47 (0.25–0.86); 0.02	-
Pre-existing coronary artery disease				
No	1	1	1	1
Yes	2.28 (1.43–3.65); 0.0006	2.87 (1.61–5.13); 0.0004	1.99 (1.03–3.89); 0.04	1.76 (1.04–2.96); 0.03
History of vascular surgery				
No	1	1	1	
Yes	2.30 (1.37–3.86); 0.002	5.28 (2.48–11.24); <0.0001	7.89 (2.34–25.60); 0.0009	
Use of other antihypertensive drugs				
No	-	-	-	1
Yes	-	-	-	2.14 (1.23–3.71); 0.007
Modifiable cardiovascular risk factors^a^				
0–2 factor	1	1	1	1
3–4 factors	4.51 (2.76–7.37); <0.0001	4.25 (2.3–7.85); <0.0001	2.84 (1.43–5.64); 0.003	2.99 (1.81–4.92); <0.0001
Referring physician				
Specialist	-	-	1	-
Family physician	-	-	0.51 (0.27–0.96); 0.04	-

^a^ Included: current smoking, diabetes, hypertension, dyslipidemia. -: indicated that the variable was not retained in the final model

## Discussion / Conclusion

In our study, 52% of patients with PAD received the recommended combined therapies for cardiovascular secondary prevention. Factors favouring the use of the combined therapies were pre-existing CAD, a history of peripheral vascular surgery and the presence of at least three cardiovascular risk factors. PAD patients presenting with critical limb ischemia, the most severe form of PAD, were less likely to receive the combined therapies. The physician referral pattern had only a minor impact on the prescription of the therapeutics.

Our study has some limitations. As most studies assessing the percentage of PAD patients receiving the preventive therapies, our study did not exclude patients who had contraindications [12–14,22–24]. This could have contributed to underestimate the proportions of PAD patients receiving the recommended cardiovascular therapies in most study, including our own. However, this decision allowed comparisons between studies before and after the introduction of the Canadian guidelines in 2005. Exclusion of patients with contraindications can be particularly difficult. On one hand, our study showed that most of the PAD patients having relative contraindications received the preventive therapies. On the other hand, absolute contraindications, mostly related to the occurrence of adverse effects of the therapies, are relatively rare [12,15]. Based on the medical record of 6837 patients with stable claudication who underwent percutaneous interventions, Arditi et al. estimated that 8.2% had contraindication to aspirin and/or statins. In 217 patients with PAD, admitted to the Hamilton General Hospital (Canada) in 2001 for PDA treatments, data collection based on medical records showed that only 5% had contraindications to antithrombotic therapy, 4% to statins and 8% to ACEI [12]. This suggests that at least 80% of the PAD patients would be eligible to receive each or a combination of the recommended therapies.

Published studies point out a suboptimal use of the cardiovascular preventive medications in PAD patients. In 8322 patients with symptomatic PAD recruited in the REACH cohort in 2003–2004, 64% were taking statins, 82% antiplatelets and 44% ACEIs. In the Hamilton study, 31% were taking statins, 59% were on antiplatelet or anticoagulant therapy and 42% were taking ACEIs [13]. Compared to this Canadian study, conducted before the release of the Canadian guidelines, our results suggest an improvement in the prescription of the cardiovascular preventive medications in PAD patients, although this difference might also be due to different clinical settings. Between 2000 and 2007, in a cohort of 34 160 Danish patients with PAD, the use of any antiplatelet therapy doubled (from 29% to 59%) and there was a 6-fold increase in the use of statins (9% to 56%)[14]. Overall, these data suggest an improvement with time of the administration of the preventive therapies among patients with PAD.

Few studies have identified the factors associated with the use of the recommended medication using multivariate analyses. In a large cohort study, taking into account others potential confounders, patients with PAD alone were less likely to use any cardioprotective agent (statins, antiplatelets, ACEI) than PAD patients with concomitant CAD (P< .0001) [14]. A cohort study conducted among 1 357 PAD patients showed that factors mostly associated with a higher utilization of statin and aspirin were pre-existing CAD (OR = 3.65; 95% CI: 2.97–4.47), peripheral revascularisation (OR = 1.42; 95% CI: 1.16–1.74) and diabetes (OR = 1.24; 95% CI: 1.03–1.49) [15]. These data suggest that physicians might be reluctant to prescribe a combination of preventive therapies in PAD patients without additional cardiovascular risk factors or diseases.

In our study, patients with critical limb ischemia were less prone than those with claudication to receive the recommended therapies, especially for statins. Patients with critical limb ischemia were older, had more comorbidity and were more often referred by emergency than those with claudication. On one hand, physicians could have questioned the long-term benefit of preventive therapies in those PAD patients with poor prognosis. On the other hand, these patients might have suffered from PAD progression because of the non-prescription of therapies such as statins and antiplatelets [25,26].

In conclusion, prescription of the recommended combined therapies in prevention of the occurrence of severe cardiovascular events is still suboptimal in patients with PAD, especially among those without concomitant cardiovascular risk factors. Additional studies could be conducted among physicians to have a better understanding of the barriers and facilitators to the application of the preventive guidelines for patients with PAD.
